# Effect of movement and developmental factors in growth and evolution in children with vesicoureteral reflux

**DOI:** 10.12861/jrip.2015.19

**Published:** 2015-09-01

**Authors:** Parsa Yousefichaijan, Fatemeh Dorreh, Mohammad Rafiei, Simin Nouri-Kopaei, Fakhreddin Shariatmadari, Abdolghader Pakniyat, Mahdyieh Naziri

**Affiliations:** ^1^Department of Pediatric Nephrology, School of Medicine, Arak University of Medical Sciences, Arak, Iran; ^2^Department of Pediatrics, School of Medicine, Arak University of Medical Sciences, Arak, Iran; ^3^School of Medicine, Arak University of Medical Sciences, Arak, Iran; ^4^Department of Pediatric Neurology, Arak University of Medical Sciences, Arak, Iran; ^5^Student Research Committee, Emergency Medicine Department, Arak University of Medical Sciences, Arak, Iran; ^6^Department of Basic Science, Clinical Research Office of Amir-Almomenin Hospital, Arak University of Medical Sciences, Arak, Iran

**Keywords:** Body weight, Vesicoureteral reflux, Child development

## Abstract

**Introduction:** Vesicoureteral reflux (VUR) is a backward flow of urine from bladder to ureter or kidney. Potential reflux is harmful because of kidney being faced with the hemodynamic high-pressure during urination. This project was carried out for high prevalence of VUR and delay in growth of children with chronic diseases. In case of growth disorder in children with this disease and its difference with healthy person, treatment can be tried by treating the growth disorder.

**Objectives:** The purpose of this study is survey of children with VUR about growth and developmental impairment.

**Patients and Methods:** All patients who performed voiding cystourethrogram (VCUG)because of UTI, divided into 2 groups, healthy and sick. History and checklist filled, patients’ height and weight measured in a standard way and ASQ questionnaires adjusted to age, used for the studying development effect. The height and weight of children measured by standard meter and scale and used the curves adjusted to age and sex. Control group entered the study with the same characteristics of case group without VUR, however, their height and weight were recorded. The way of evolution studied according to Nelsons evolution table and ASQ questionnaire.

**Results:** The both groups (total of 150 studied children) in the area of development of fine motor, gross motor and indicators of mean and percentile of height and weight and parents’ literacy, had a significant difference (*P*< 0.05). It is can be due to better assessment and follow, higher education levels and better socioeconomic situation.

**Conclusion:** Children with VUR, in terms of height and weight growth and index of gross and fine movements and communicate were better than normal children.

**Implication for health policy/practice/research/medical education:**

Height, weight, gross and fine movements and communicate growth index are better in the children with vesicoureteral reflux (VUR) than normal children. It is can be due to better assessment and follow, higher education levels and better socioeconomic situation.

## Introduction

Vesicoureteral reflux (VUR) refers to the retrograde flow of urine from bladder to ureter and kidney (1-3). It is thought that condition insufficiency due to congenital bladder and ureter junction occurs. The ureteral attachment to the bladder normally is oblique, between the bladder mucosa and detrusor muscle, creating a flap-valve mechanism that prevents reflux (4-7). Reflux occurs when the submucosal tunnel between the mucosa and detrusor muscle is short or absent (8,9).

Reflux is usually congenital and occurs familial in 1% of children. Urinary reflux predispose kidney to infections (pyelonephritis) by crossing bacteria from bladder to the upper urinary tract. Inflammatory reaction caused by a kidney infection can lead to damage and create scarring in kidney. The renal damage associated with reflux, called nephropathy reflux (10,11). Kidney damage of febrile urinary tract infections in children with reflux is 3 times more likely than children without reflux (12-14). Severe kidney damage impairs kidney function and can lead to high blood pressure through renin and renal failure, end-stage renal disease (ESRD), somatic growth retardation and increased incidence of disease in pregnancy (15,16).

Reflux commonly exists in 25% of children with neuropathic bladder at birth. Similarly occurs in many cases in patients with myelomeningocele, agenesis sacral and anal atresia. Reflux can be seen in 50% of patients with posterior valve urine (4-8). If reflux is associated with increased pressure within the bladder (so that can be seen in dysgenesis sphincter–detrusor or in obstruction of bladder outlet), this can lead to kidney damage even in the absence of infection.

Reflux is usually known and discovered during the investigation of a urinary tract infection. Around 80% of the children are girls and their mean age at diagnosis is 2-3 years. To evaluate urinating abnormalities, renal failure, high blood pressure or other pathological processes of urinary tract, in the other children during doing VCUG (voiding cystourethrogram) reflux diagnosed. Also primary reflux can be diagnosed during evaluation of hydronephrosis during the prenatal period, in this population, 80% were male children and level of reflux usually diagnosed higher than girls (1-8).

The goal of treatment is to prevent pyelonephritis, kidney damage due to reflux and other complications. The American urological association suggests surgical treatment in children who failed their drug treatment approach (occurrence of recurrent urinary tract infection, persistent reflux) or those with high levels of reflux and consequently their spontaneous recovery is unlikely (4-10).

## Objectives

Given the high prevalence of urine back from bladder to ureter and kidney and possible impairment of growth and development, the purpose of this study is survey of children with VUR in Arak city.

## Patients and Methods

This is a case-control study which conducted on 150 children with urinary tract infection who have indications of ultrasound and VCUG referred to the pediatric clinic in Arak in 2012-2013. Children were divided into 2 groups, with VUR and healthy (normal VCUG). Seventy-five of these children were with VUR (case group) and 75 with normal VCUG were healthy (control group). The height and weight of patients measured using standard methods (1) and to study the evolution of age-appropriate ASQ questionnaire (age and stages questionnaires) were used. ASQ questionnaire used to assess growth and development in children under 6 years. The children’s height and weight were measured by a standardized scales and meters and to prevent systematic and random errors the curves adjusted to age and gender. Height and weight were recorded for both groups and manner of development according to the ASQ questionnaire investigated. In an ASQ questionnaire (indicators of social development, problem solving, a big, fine motor, communication) diagnosed defects if a child acquired less than 31.5 developmental scores and a child diagnosed healthy if the score is greater than 31.5 (1,2).

### Ethical issues

1) The research followed the tenets of the Declaration of Helsinki; 2) informed consent was obtained, and they were free to leave the study at any time and 3) the research was approved by the ethical committee of Arak University of Medical Sciences.

### Statistical analysis

Describing the data to determine frequencies, the descriptive statistical methods were used. The one-way analysis of variance test (ANOVA) used to analysis data. In all design process, ethical considerations such as informed consent of participation in the plan and exclusion is optional and maintain the confidentiality of the obtained information, were taken into consideration. Data were analyzed by SPSS version 19 software. In this study, *P* ≤ 0.05 was considered as significant.

## Results

The present study examined 150 children aged 1-6 years, that in case group 18.9% of fathers had diploma, 81.1% had college education, while in the control 67.6% had diploma, 32.5% had college education that statistically, fathers’ education index between the 2 groups showed a significant difference (*P *= 0.001). The mothers’ literacy rate in the case was 6.8% below the diploma, 83.8% diploma, and 9.5% with college education, while in the control group 2.7% had below the diploma, 17.6% diploma, 79.7% with college education which statistically mothers’ literate index, there was a significant difference between the groups (*P *= 0.002). The average difference between the height and weight percentiles in the 2 groups were significantly different (*P *< 0.05). In the case the mean height percentile rank has been 2.7 nearly 15-50 and in the control 2.01 means 3-15. There was no significant relationship in the groups, based on the frequency of classification of parents present index (*P *= 0.9). In the focus group 64% of persons with poor social indicators and 36% with appropriate social indicators were estimated. While in the other 45.3% estimated with poor social indicators and 54.7% were with appropriate social indicators which in developmental fields of social indicators there was no significant difference in the groups (*P *= 0.3).

In terms of the communication field index, in the focus group 25.3% of persons estimated poor and 74.7% were appropriate, while in the control group 41.3% estimated poor and 58.7% were appropriate which in the terms of the evolutionary field of communication there was no significant difference in the groups (*P *= 0.061). In the terms of economic indicators there was not a significant relationship in the groups (*P *= 0.4) and in terms of weight percentile index, there was a significant relationship (*P *= 0.49; Table 1).

**Table 1:** Frequency distribution table in the 2 groups based on the obtained scores of effective factors in the growth and development

		**Case**	**Control**	**Total**	***P*** ** value**
Social index	Less than 31.5 (inappropriate)	27 (36.0)	34 (45.3)	61 (40.7)	0.3
	More than 31.5 (appropriate)	48 (64.0)	41 (54.7)	89 (59.8)	
Problem solving index	Less than 30.5 (inappropriate)	40 (53.3)	39 (52.0)	79 (52.7)	0.8
	More than 30.5 (appropriate)	35 (46.7)	36 (48.0)	71 (47.3)	
Fine motor index	Less than 30.5 (inappropriate)	24 (32.0)	40 (53.3)	64 (42.7)	0.013
	More than 30.5 (appropriate)	51 (68.0)	35 (46.7)	86 (57.3)	
Gross motor index	Less than 32.7 (Inappropriate)	19 (25.3)	37 (49.3)	56 (37.3)	0.001
	More than 32.7 (appropriate)	56 (74.7)	38 (51.7)	94 (62.1)	
Communication evolutionary index	Less than 31.7 (inappropriate)	19 (25.3)	31 (41.3)	50 (33.3)	0.061
	More than 31.7 (appropriate)	56 (74.7)	43 (57.3)	99 (66.0)	

In the case group, in the terms of problem solving field index 53.3% persons estimated inappropriate and 46.7% were appropriate. While in the control group 52% estimated inappropriate and 36% were appropriate which there was not a significant difference in the terms of problem solving evolution field (*P *= 0.8). Considering fine motor index in cases 32% were inappropriate and 68% estimated appropriate. While in the control 53.3% were inappropriate and 46.7% were appropriate which there was a significant difference between the groups in the evolution field (*P *= 0.013). Considering index of gross motor in the case 25.3% estimated inappropriate and 74.7% were appropriate. While in the control group 49.3% estimated inappropriate and 51.7% were appropriate which there was a significant difference in the evolution field (*P *= 0.001; Table 1).

## Discussion

Considering the evolution field of social indicators and problem solving in the groups there was no significant difference between cases and controls. Considering the evolution field of fine motor indicators in the both groups, differences arising from problems was in the area of the fine motor in the control group. Also in the area of gross motor index, difference between the groups was the result of problems in the field of gross motor in the control group. In the communication and economic indexes, no significant difference was in the case and control groups. In this study in terms of weight index, weight significantly in the normal group was less than patient group. In this study in terms of height index, height significantly in the healthy group was less than patient group. On the basis of father and mother educations there was a significant difference in both groups. In a survey conducted in Kermanshah by Malaki et al, on 106 children less than 5, the results showed in children with urinary tract infection (UTI) and normal glomerular filtration and reflux with any degree of intensity and time, there is no negative effects on growth index which contrasts with the present study. The reason can be found in higher sample volume in present study than the above (3). In Das et al study, which was conducted on 10 children 1-10 years with VUR, height index was lower than that of other children. The general index of weight relative to height increased which in the present study does not comply with the height, which could be due to the higher number of cases examined in the study (4). In a study of 108 children with UTI, in which VUR was diagnosed before age 11, done by Fu et al, it was shown that early treatment of reflux can reduce the negative effects of reflux on height loss, especially if it does not result in antibiotic prophylaxis treatment (11). Baquedano Droquett et al (6), studied 85 children with UTI, it was shown UTI with or without reflux and with or without kidney damage except chronic renal failure (CRF) does not have a negative effect on the patient physical growth which it does not conflict with the present study. Polito et al (7), studied 32 patients with VUR, it was shown patients with VUR diagnosed during fetal life and have normal glomerular function, if during the first year of life to be treated with antibiotic prophylaxis, they have sufficient physical growth. In other study of Polito et al, on 94 patients with VUR performed before puberty, after VUR treatment (in a medical treatment group and a surgical treatment group) it was observed that the height and weight index for height increased in the first year of treatment and the rate of increase was higher than the second year (8). In another study of Polito et al, performed on 156 children with VUR was found patients with VUR and without renal scarring compared to height with control group did not much differ in height and weight. However, patients with VUR and renal scarring in height and weight index for height is lower than the control group which does not compatible with the present study (9). The study of Smelli et al showed that long-term prophylaxis with low-dose co-trimoxazolehas do not effect on somatic growth in patients with urinary tract infection with or without reflux (10). Wingen et al (12), studied 306 children younger than 11 years with VUR, grade III and IV, VUR had no effect on the growth rate and the only factor that had an impact on the growth rate was age of onset of VUR which was not inconsistent with the present study (11). In Sutton and Atwell study, children growth before and after surgery of the back urinary from the ureter into the bladder was assessed. Growth in children after a successful surgery was significantly better (16). Additionally, Baquedano Droquett et al (6) presented a study which showed there was not difference in children with VUR and normal subjects.

## Conclusion

Height, weight, gross and fine movements and communicate growth index are better in the children with vesicoureteral reflux (VUR) than normal children. Also the mean difference of percentile for height and weight and parents’ education is better. It is can be due to better assessment and follow, higher education levels and better socioeconomic situation. This could be a sign of better assessment and follow-up of parents with higher education levels and better socioeconomic status.

## Limitations of the study

There was the limitation of working parents to fill out the questionnaire but after explaining to parents about effect of disturbance on growth and evolution they agreed to cooperate.

## Acknowledgements

We appreciate the Research Council and Vice Chancellor for research of University of Arak Medical Sciences, who were responsible for funding the project, and also the parents and children who worked on this project.

## Authors’ contribution

All authors participated equally in the project. YP; funding of patient with VUR. DF and AGP; evaluation of growth. SF; evaluation of development. RM and Naziri M; evaluation of biostatic.

## Conflicts of interest

The authors declared no competing interests.

## Ethical considerations

Ethical issues (including plagiarism, data fabrication, double publication) have been completely observed by the authors.

## Funding/Support

Our study was funded by the Student research committee, Arak University of Medical Sciences (grant No. 742).
